# Reply to Hertig-Godeschalk et al. Comment on “Bai et al. Effect of Eccentric Training with Different Durations, Intensities, and Contraction Velocities on Upper Limb Muscle Strength: A Meta-Analysis. *Life* 2025, *15*, 456”

**DOI:** 10.3390/life15121824

**Published:** 2025-11-28

**Authors:** Zhe Bai, Dong Zhang, Dongxue Liang, Xiaoke Chen, Xinyu Shi, Shu Chen

**Affiliations:** 1 Institute of Artificial Intelligence in Sports, Capital University of Physical Education and Sports, Emerging Interdisciplinary Platform for Medicine and Engineering in Sports, Beijing 100191, China; 2 Department of Physical Education, Tsinghua University, Beijing 100084, China

We sincerely appreciate your valuable feedback on our manuscript, particularly your concerns regarding the control group designs addressed in the comment by Hertig-Godeschalk et al. [1]. We fully understand your concern that the control groups in these studies may differ from our original inclusion criteria (which required control groups to undergo concentric training or be inactive). After careful consideration, we believe that directly removing these four articles would reduce the sample size from 11 to 7, potentially leading to insufficient statistical power for our analysis and affecting the representativeness of our results. As demonstrated in our sensitivity analysis, two highly heterogeneous articles have already been excluded. Further reduction in the number of articles could introduce unintended bias. Therefore, we propose a slight adjustment to our inclusion criteria to integrate the existing evidence in a more inclusive manner while maintaining scientific rigor. The following outlines our proposed modifications and the rationale behind them.

We propose the following modification to the third inclusion criterion in Section 2.2 [2] of the original manuscript: Studies must provide comparative data that can be used to calculate effect sizes, which can take the form of traditional control groups (e.g., a concentric training group or inactive group); single-group pre–post design, provided that the data allows for the calculation of standardized mean difference (SMD); or other forms of control (e.g., baseline comparison based on different outcome measures), as long as they can reasonably reflect the effects of eccentric training.

Regarding the appropriateness of the control groups in the four aforementioned studies, the specific explanations are as follows:

Scharer et al. [3] and Scharer et al. [4] both used static actions as controls, aiming to evaluate static exercise performance under slow eccentric training. Both groups showed significant improvements in post-testing. In the field of sports science, single-group pre–post or specific action controls are common in elite athlete studies or exploratory designs. These types of controls actually reflect real training contexts and do not constitute a methodological flaw.

The control group in Vetter et al. [5] was the concentric peak torque of the rotator cuff external rotators at 180°/s. In Table 2 of this article, this can be considered an internal control within the same muscle group [5]. This metric provides direct effect size data through pre- and post-testing comparisons, and peak torque, as a direct parameter of force output, is highly correlated with the outcome measures of interest in this study.

In Perret et al. [6] study, the control group used grip strength as a functional indicator of overall upper limb strength. Grip strength, like exercises such as the bench press, belongs to the upper limb muscle strength assessment system. The two are consistent in reflecting the function of synergistic muscle groups in the upper limb; therefore, it is reasonable to use grip strength as a control.

The overall effect size and subgroup analyses in our study have shown significant results. We believe that appropriately relaxing the inclusion criteria will not introduce significant heterogeneity; rather, it will help maintain the completeness of the evidence chain and the practical significance of the analysis.
